# Cost and time-efficient construction of a 3′-end mRNA library from unpurified bulk RNA in a single tube

**DOI:** 10.1038/s12276-024-01164-8

**Published:** 2024-02-27

**Authors:** Jungwon Choi, Jungheun Hyun, Jieun Hyun, Jae-Hee Kim, Ji Hyun Lee, Duhee Bang

**Affiliations:** 1 Department of Chemistry, Yonsei University, Seoul, Republic of Korea; 2 Department of Clinical Pharmacology and Therapeutics, College of Medicine, Kyung Hee University, Seoul, Republic of Korea; 3 Department of Biomedical Science and Technology, Kyung Hee University, Seoul, Republic of Korea

**Keywords:** RNA sequencing, Next-generation sequencing

## Abstract

The major drawbacks of RNA sequencing (RNA-seq), a remarkably accurate transcriptome profiling method, is its high cost and poor scalability. Here, we report a highly scalable and cost-effective method for transcriptomics profiling called Bulk transcriptOme profiling of cell Lysate in a single poT (BOLT-seq), which is performed using unpurified bulk 3′-end mRNA in crude cell lysates. During BOLT-seq, RNA/DNA hybrids are directly subjected to tagmentation, and second-strand cDNA synthesis and RNA purification are omitted, allowing libraries to be constructed in 2 h of hands-on time. BOLT-seq was successfully used to cluster small molecule drugs based on their mechanisms of action and intended targets. BOLT-seq competes effectively with alternative library construction and transcriptome profiling methods.

## BOLT-seq: The future of high-speed, low-cost transcriptome profiling

In the biomedical research field, RNA sequencing is a valuable tool. However, the standard method of preparing samples for RNA-seq is expensive and time-consuming, particularly when many samples are required. This study, led by Dunhee Bang, presents a new technique named Bulk transcriptOme profiling of cell Lysate in a single pot. BOLT-seq simplifies the RNA-seq procedure by using fewer steps and homemade reagents, significantly reducing both cost and time. BOLT-seq is an experiment where up to 1000 cells are processed directly in one tube without purifying the RNA first. The researchers concluded that BOLT-seq is a significant advancement for large-scale genetic studies. In the future, this method could make understanding how different treatments affect gene activity in cells easier and more affordable, potentially speeding up drug discovery and personalized medicine.

This summary was initially drafted using artificial intelligence, then revised and fact-checked by the author.

## Introduction

RNA-seq is used routinely in biomedical research and can be readily performed using commercially available kits^1,2^. Although the cost of DNA sequencing has substantially decreased over time, the cost and complexity of standard library preparation remain significant concerns^3–5^. The main steps performed to construct libraries from bulk transcriptome profiling through RNA-seq include RNA extraction, reverse transcription, second-strand complementary DNA (cDNA) synthesis, tagmentation, and PCR amplification. Typically, each of these sequential reaction steps is performed using a commercial kit or a set of specific reagents and must be properly completed before the next step is performed^6–8^. After each experimental step, intermediate products must be purified and verified. Even though the cost of sequencing has decreased, the cumulative labor, time, and cost needed to construct NGS libraries using commercially available kits can be prohibitive many libraries are needed. Because the costs of preparing NGS libraries and sequencing increase with increasing sample numbers, financial considerations often limit the choice of experimental design.

Through whole RNA-seq-based construction of NGS libraries, transcriptomes can be thoroughly analyzed. However, these protocols are usually associated with longer preparation times and increased costs^9,10^. On the other hand, 3′-end mRNA-seq, which limits DNA sequence analysis to the 3′-terminal region of mRNA transcripts, is an attractive approach for increasing throughput^11^. Furthermore, more samples can be combined per NGS run, and more relative read depth per gene can be achieved with 3′-end mRNA-seq than with whole transcriptome sequencing. To exploit these advantages, several 3′-end mRNA-seq library preparation protocols as well as analytical procedures have been developed.

Several newly developed 3′-end mRNA-seq protocols require smaller amounts of reagents and fewer commercial kits, resulting in reduced costs. For example, Bulk RNA Barcoding and sequencing (BRB)-seq is a rapid, inexpensive method for generating 3′-end mRNA-seq libraries from bulk RNA^12^. BRB-seq uses “in-house”-produced Tn5 transposase for tagmentation of double-stranded cDNA, resulting in a substantial reduction in cost. However, in the BRB-seq protocol, RNA must be purified using a commercially available kit. Then, purified RNA is used for reverse transcription for the next reaction step. On the other hand, Digital RNA with pertUrbation of Genes (DRUG)-seq is a unique 3′-end mRNA-seq method that uses bulk RNA in cells that are directly lysed in mild lysis buffer in a 96-well plate format^13^. Bulk RNA in the lysate is then individually reverse-transcribed and pooled prior to purification. This process minimizes hands-on time and work at the start of the procedure; thus, multiple samples can be processed simultaneously. However, DRUG-seq requires sample pre-amplification and multiple purification steps, making it a slower and more labor intensive method (Supplementary Fig. 1).

Here, we present Bulk transcriptOme profiling of cell Lysate in a single poT (BOLT-seq), a new 3′ mRNA-seq method that incorporates some features of BRB-seq and DRUG-seq. BOLT-seq is a highly scalable cost-effective transcriptome sequencing method targeting 3′-end sequences of unpurified bulk mRNA directly in the lysate of up to 1000 cells; BOLT-seq also uses reverse transcriptase (RT)^14^ and Tn5 transposase^15^ purified in our laboratory (in-house) as well as in-house prepared reaction buffers to reduce experimental cost. In the BOLT-seq protocol, purification of intermediate products is not necessary until the library amplification step; therefore, the experiment be carried out in a single tube. By subjecting RNA/DNA hybrid duplexes to Tn5 transposase-mediated tagmentation^16^, second-strand cDNA synthesis and pre-amplification can be omitted from the BOLT-seq protocol. BOLT-seq requires minimal hands-on labor, and a 3′-end mRNA library can be constructed in less than one day for under US $1.40 per sample (excluding the cost of NGS sequencing) [Supplementary Table 1] while fulfilling standard expectations for the quality of DNA sequence and gene expression data in multiple cell lines. To demonstrate the potential of the method, BOLT-seq was used to analyze the effects of 35 small molecule drugs on the transcriptome of cells in a 96-well microtiter plate. The data from this experiment were successfully used to cluster the drugs by their mechanisms of action and intended targets.

## Materials and methods

### Cell lines

We purchased the HEK293T, A549, and NIC-H358 cell lines from the Korean Cell Line Bank (KCLB No. 21573, KCLB No. 10185, KCLB No. 25807). HEK293 cells were maintained in DMEM or RPMI Medium 1640 (Gibco, USA) with 10% fetal bovine serum (Gibco, USA) and 1% penicillin/streptomycin (Gibco, USA). Cells were cultured at 37 °C under 5% CO_2_.

### Purification of reverse transcriptase and Tn5 transposase

The reverse transcriptase used in this study was a variant of M-MuLV RT cloned and inserted into the pET-28a(+) vector (Millipore Sigma, USA), which carries a kanamycin-resistance gene. M-MuLV was expressed and purified according to the protocol published by Baranauskas et al.^12^. The Tn5 transposase used in this study was from pTXB1-Tn5, which expresses Tn5 transposases and an ampicillin resistance gene [gift from Rickard Sandberg (Addgene plasmid #60240; http://n2t.net/addgene:60240; RRID: Addgene_60240)]. Tn5 transposase was expressed and purified according to the protocol published by Picelli et al.^13^.

### RNA-seq library preparation

Cells were cultured in 96-well microtiter plates, and total RNA was purified using an RNeasy mini kit (Qiagen, Netherlands). Total RNA quality was analyzed using the 4150 TapeStation System (Agilent, USA). NEBNext Ultra II RNA libraries were prepared from 200 ng purified total RNA according to the manufacturer’s instructions (NEB, USA). Libraries were sequenced by NovaSeq 6000.

### BOLT-seq library preparation

To prepare BOLT-seq libraries, 96-well plates were seeded with 10,000 cells, incubated overnight, washed twice with DPBS (Gibco, USA), and lysed in 60 µL of lysis buffer containing 0.3% IGEPAL CA-630 (Sigma Aldrich, USA). The plates were incubated for 30 min with shaking at 800 RPM. Cell lysate (6 µL) was transferred into a PCR tube for library preparation. RNA was denatured by adding 1 µL RT-Mix-A containing 10 µM anchored oligo(dT)30-P7 RT primer (Integrated DNA Technologies, South Korea) and 1 µL 1 mM dNTP mix (Thermo Fisher, USA) to the cell lysate. Then, the mixture was incubated at 65 °C for 5 min and quickly cooled on ice for 3 min. The RT reaction was performed by adding 7 µL RT-Mix-B containing 117 mM Tris-HCl (pH 8.3), 175 mM KCl, 7 mM MgCl_2_, 23 mM DTT, 23% PEG8000 (w/v) (bioPLUS, USA), 5 U RNase OUT Ribonuclease inhibitor (Thermo Fisher, USA), 0.5 µL in-house purified M-MuLV RT and incubation at 50 °C for 60 min. The reaction was inactivated by incubation at 80 °C for 10 min. No purification step was needed. DNA/RNA hybrid duplexes were subjected to tagmentation by adding 5 µL of TD-Mix containing 40 mM Tris-HCl (pH 7.5), 20 mM MgCl_2_, 30% PEG8000 (w/v) (bioPLUS, USA), 20% tetraethylene glycol (Thermo Scientific, USA), and 0.5 µL of in-house purified Tn5 transposase and incubated at 55 °C for 30 min. Tagmentation was stopped by adding 5 µL of 0.2% SDS. No purification step was needed. Gap-filling and PCR amplification were performed by adding 25 µL PCR-Mix containing 5x HiFi Fidelity Buffer (Kapa Biosystems, UK), KAPA dNTP Mix (Kapa Biosystems, UK), KAPA HiFi HotStart DNA Polymerase (Kapa Biosystems, UK), 0.5 µL in-house purified RT, and 2 µL NGS indexed primers. Gap-filling and PCR were performed as follows: 50 °C for 10 min for gap-filling, 95 °C for 3 min for initial denaturation and inactivation of RT, 18 cycles of 95 °C for 30 s, 60 °C for 30 s, 72 °C for 30 s, 72 °C for 3 min for final extension. The indexed products were purified at 0.6X with SpeedBead Magnetic Carboxylate Modified Particles (GE Healthcare, UK). Library products were quantified with Qubit 2.0, and library length distribution was analyzed with the 4150 TapeStation System (Agilent, USA).

### RNA-seq library data analysis

Raw NGS reads were trimmed to remove low-quality bases and adapter sequences using Trimmomatic 0.39^17^ (ILLUMINACLIP1:30:7, LEADING:3, TRAILING:3 SLIDINGWINDOW:4:15 MINLEN:20). Subsequent processes, including trimming, paired-end, and single-end options, were applied according to RNA-seq methods. Trimmed reads were mapped to the human genome (GRCh37/hg19) using STAR 2.7.6a^18^ (--outSAMmapqUnique 60). Only uniquely mapped aligned reads were selected and used for subsequent analyses. bam files were subsampled identically in each experiment. The subsampling size is specified in the figure legend as needed. SAMtools 1.11^19^ was used to sort uniquely mapped reads and for subsampling. Unique subsampled mapped reads were assigned to the set of 42149 annotated genes in the GRCh37 assembly of the human genome using featureCount 1.6.5^20^ from the package Subread. Raw read counts from HEK293T and A549 cells were analyzed with DESeq2^21^ to detect DE genes.

### Gene set expression analysis

To test the GSEA of the H358 drug reaction performed with the NEBNext Ultra II RNA kit and BOLT-seq, a KRAS dependency gene-related gene set of previously published data^22–26^ was obtained. Enrichment of gene sets was analyzed using GSEA v3.0^27^ and the MSigDB gene set database from published data^28^ for the Hv6.0 collection of hallmark sets, the C2v6.0 collection of chemical and genetic perturbations (CGP) and canonical pathways (CP), the C5v6.0 collection of GO biological and molecular functions, and the C6v6.0 collection of oncogenic signatures.

## Results

### Bulk transcriptome profiling of cell lysate in a single pot—BOLT-seq library preparation and workflow

**Fig. 1:**
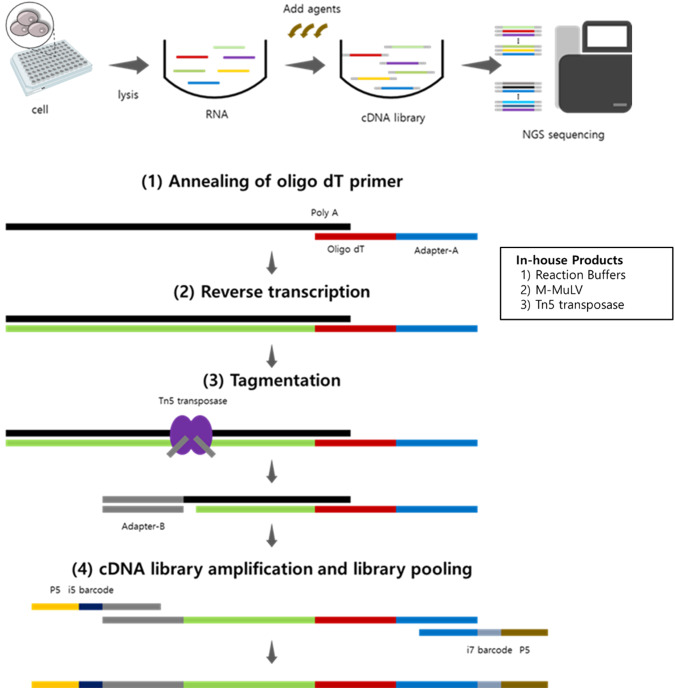
Overall BOLT-seq scheme. Bulk transcriptOme profiling of cell Lysate in a single poT—BOLT-seq. The steps of BOLT-seq are shown schematically. Sequential reactions were carried out in a single well of a 96-well microtiter plate with no intermediate purification steps. After the final library amplification step, products are purified from each well and pooled as desired.

As a method for gene expression profiling, BOLT-seq is a streamlined, inexpensive, and time-efficient; utilizes a 96-well microtiter plate format; does not require intermediate purification steps; and does not involve commercially available kits. The workflow and sequential steps of BOLT-seq library preparation are shown schematically in Fig. 1. To summarize, after cells were lysed in the well of a 96-well plate, reverse transcription was performed using in-house purified Moloney murine leukemia virus (M-MuLV) reverse transcriptase and bead-anchored oligo-dT primers [Supplementary Table 2]. Note that a substantial cost reduction was achieved by using in-house purified M-MuLV and in-house prepared reaction buffers. To ensure optimal RT efficiency, the activity of in-house purified M-MuLV RT was tested with different reaction buffers^14^. After the RT step, RNA/DNA hybrid duplexes were used for the Tn5 transposase-mediated tagmentation reaction; thus, second-strand cDNA synthesis could be omitted^16^. As the BOLT-seq protocol eliminates the step in which RNA transcripts are converted to double-strand DNA, the experimental cost and the time needed for library preparation are also reduced. Further cost reduction was achieved by purifying Tn5 transposase and preparing its reaction buffer in-house according to a protocol published by Picelli et al.^15^. The products of Tn5 transposase-mediated tagmentation were then used without purification in the subsequent gap-filling and PCR amplification steps. Thus, BOLT-seq is a streamlined procedure that reduces the total time for NGS library construction to 4 h at a dramatically reduced cost to US $1.40 per well.

### Performance of BOLT-seq

To readily compared the method to traditional RNA-seq methods, BOLT-seq was performed under several different experimental conditions. Initially, the variables tested included the number of input cells per well and the presence or absence of a crowding agent, such as polyethylene glycol 8000 (PEG8000). HEK293T and A549 cell lines were used to compare each experimental condition. To test the robustness of BOLT-seq, initial experiments were performed using an appropriate number of cells per well, and in-house purified Tn5 transposase was used for RNA/DNA hybrid tagmentation. Finally, the index PCR cycle was fixed at 18 cycles for comparison.

As reported, the efficiency of the previous protocol decreased when >1000 cells were lysed in a single well^29^. Therefore, the BOLT-seq libraries prepared in this study were derived from no more than 1000 cells per well, and the performance of BOLT-seq using 100, 500, and 1000 cells per well was compared. For libraries derived from 100 cells, many NGS sequencing reads had to be discarded due to unacceptably short length (Supplementary Fig. 2a); as a result, no additional experiments were performed using 100 cells per well. More genes were detected in libraries prepared from 1000 cells than in libraries prepared from 500 cells, but this difference was not significant (Supplementary Fig. 2b). For consistency and comparability of reaction controls, all subsequent experiments in this study used 1000 cells per well.

PEG8000 is frequently added to RT reactions to increase molecular crowding, but it has also been used to effect conformational change and facilitate Tn5 transposase-mediated tagmentation of RNA/DNA hybrid duplexes^16^. In this study, no significant difference in gene detection was observed when BOLT-seq was performed in the presence of 0% or 9% PEG8000 (Supplementary Fig. 2c). However, a significantly higher DNA yield was obtained during the final PCR stage of BOLT-seq in the presence of 9% PEG8000. Therefore, all subsequent BOLT-seq reactions were performed in the presence of 9% PEG8000.

**Fig. 2:**
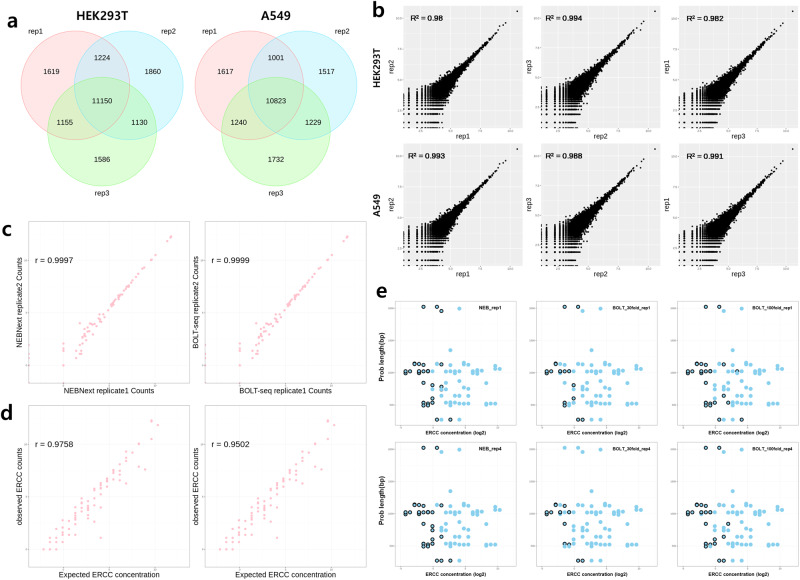
Performance of BOLT-seq. Reproducibility of the BOLT-seq method All experimental replicates were subsampled to 1 M. Venn diagrams in (**a**) represent overlapping subsets of genes detected in each of three replicate BOLT-seq samples using 1000 HEK293T (left) or A549 (right) cells per well in a 96-well microtiter plate. **b** Normalized gene read counts from each of the three replicate experiments per cell line shown in (**a**) were compared pairwise. Correlation values (r) for each pairwise comparison are shown in the upper left corner of each panel. Upper panels, HEK293T cells; lower panels, A549 cells. **c** Correlation between unique normalized ERCC read counts in two experimental BOLT-seq replicates using 1000 HEK293T cells. Left panel, NEBNext; right panel, BOLT-seq. **d** Correlation between observed and expected ERCC read counts using NEBNext (left) or BOLT-seq (right). **e** The relationship between the ERCC concentration and probe length using NEBNext (left), BOLT-seq 1/30 (middle), and BOLT-seq 1/100 (right). Undetected dropouts are indicated by a black circle.

The reproducibility of BOLT-seq was evaluated by comparing the number of expressed genes detected in common between three independent replicate experiments in two different cell lines. The results showed that 11,150 genes were detected in all three replicates in HEK293T cells, and 10,823 genes were detected in all three replicates in A549 cells, representing 56.5% and 56.4% of all genes detected in HEK293T and A549 cells, respectively (Fig. 2a). In addition, normalized gene read counts were compared pairwise for all three replicate experiments in HEK293T and A549 cells, and correlation values ranged from 0.980 to 0.994 in HEK293T cells and from 0.988 to 0.993 in A549 cells (Fig. 2b).

BOLT-seq data were also verified using variable amounts of a spike-in reference standard and methods developed by the US National Institute of Standards and Technology-sponsored External RNA Controls Consortium (ERCC). For each experimental replicate in HEK293T and A549 cells, ERCC read counts were compared with expected ERCC values, and BOLT-seq data were compared with data from the NEBNext Ultra II RNA Kit. The results showed that normalized ERCC counts between replicates were highly correlated (*r* > 0.999) (Fig. 2c, Supplementary Fig. 3a) for the BOLT-seq and NEBNext methods, indicating that both methods achieve a high level of reproducibility. The observed ERCC counts and expected ERCC amounts were also highly correlated (Fig. 2d, Supplementary Fig. 3b), with correlation values ranging from 0.942 to 0.976 for NEBNext and from 0.924 to 0.950 for BOLT-seq. These results confirm that BOLT-seq achieves an acceptable and desirable level of performance. Transcript detection efficiency was also evaluated by plotting the ERCC probe concentration and length using the RNA-seq method; the results indicate that selective sequencing does not occur as a function of probe length. The results also show dropout, which is depicted as black circles in Fig. 2e, at low ERCC probe concentrations independent of the ERCC probe length. For replicates with BOLT-seq, the ERCC probe mix was spiked at ratios of 1:30 or 1:100 to demonstrate that low-concentration ERCC probes could be better detected and sequenced in samples prepared at a ratio of 1:30 than 1:100. These results indicate that variations in probe length or concentration are not associated with sequencing bias during BOLT-seq. Thus, compared to traditional mRNA-seq methods, BOLT-seq is simpler, less labor intensive, and less expensive.

### Characterization of M-MuLV reverse transcriptase purified in-house

BOLT-seq is performed with in-house purified M-MuLV RT instead of commercially available RT to lower the cost of library preparation. The quality of in-house purified M-MuLV RT was established by comparing the number of DE genes detected by BOLT-seq with in-house M-MuLV RT, commercially available Maxima™-H RT, or SuperScript™ IV (SSIV) RT. These data were also compared with data generated with NEBNext. Venn diagrams representing the number of DE genes detected in each condition are shown in Supplementary Fig. 4a. The results show that in-house M-MuLV RT detects fewer DEs than SSIV RT, but it also detects more DEs than Maxima™-H RT. Therefore, while the performance of in-house M-MuLV RT is not optimal, it performs comparably to commercially available preparations of M-MuLV RT. We also compared the log2-fold change in DE genes with BOLT-seq or NEBNext (Supplementary Fig. 4b), which revealed that the results with BOLT-seq and NEBNext (*r* = 0.961) and the log2-fold change (lfc) observed with BOLT-seq or NEBNext using in-house M-MuLV RT are also highly correlated (*r* = 0.944). These results suggest that the function and activity of in-house M-MuLV RT are comparable to the function and activity of commercially available RT preparations. Thus, in-house M-MuLV RT can be safely used without compromising data quality while considerably reducing the cost of library construction with BOLT-seq.

### Comparing BOLT-seq to other RNA-seq methods

**Fig. 3:**
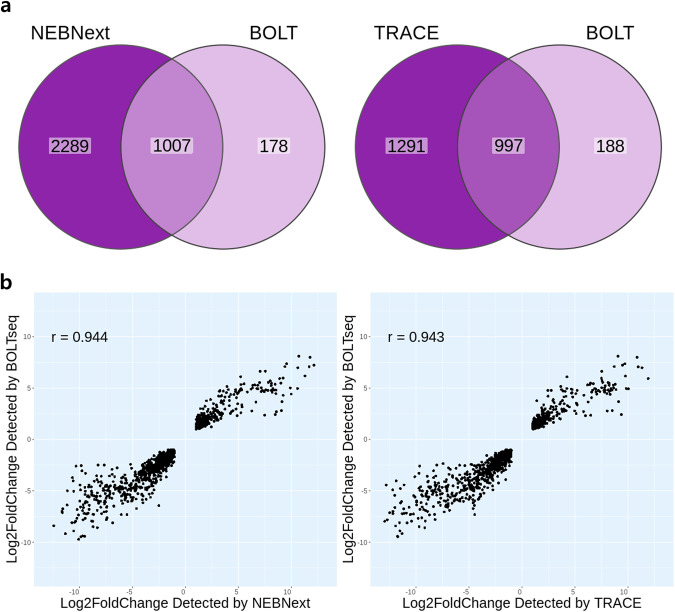
Comparison between BOLT-seq and the canonical RNA-seq method. Comparison between BOLT-seq and the canonical TRACE-seq and NEBNext methods. All replicates were subsampled to 1 M (replicates: NEBNext *n* = 3, TRACE-seq *n* = 3, BOLT-seq *n* = 3). **a** Venn diagrams show overlapping subsets of DE genes detected with NEBNext and BOLT-seq (left) or TRACE-seq and BOLT-seq (right). NEBNext and TRACE-seq were performed using 200 ng of total RNA purified from HEK293T or A549 cells. BOLT-seq was performed using the lysates of 1000 HEK293T or A549 cells. **b** Correlation between the log2-fold change (lfc) of DE genes detected by NEBNext and BOLT-seq (left) or TRACE-seq and BOLT-seq (right). Cutoff criteria were threshold: |lfc| > 1, p-adj <0.05).

Next, the performances of BOLT-seq, TRACE-seq^30^, and NEBNext were compared. For these experiments, 200 ng of total RNA was purified from HEK293T and A549 cells and used for library construction with TRACE-seq or NEBNext, whereas BOLT-seq was performed using 1000 HEK293T or A549 cells per well. For each experimental method, DE genes were identified by comparing HEK293T and A549 cells using DESeq2. A *p* value less than 5 × 10^6^ and |log2-fold change| > 1 were used as cutoff criteria, and the results are represented in the Venn diagrams in Fig. 3a. TRACE-seq and NEBNext detected 2288 and 3296 DE genes, respectively, while 1007 DE genes were detected by both NEBNext and BOLT-seq, representing 85% of all DE genes detected by BOLT-seq (Fig. 3a, left). Similarly, 997 DE genes were detected by both TRACE-seq and BOLT-seq, representing 84.1% of all DE genes detected by BOLT-seq (Fig. 3a, right). Although BOLT-seq detects far fewer DE genes than canonical RNA-seq methods, >80% of the DE genes detected by BOLT-seq are also detected by TRACE-seq and NEBNext. In addition, the lfc values of DE genes detected by NEBNext or TRACE-seq and BOLT-seq were highly correlated (*r* > 0.94 for both; Fig. 3b, left and right). Therefore, the reproducibility and performance of BOLT-seq and established RNA-seq methods are comparable.

### Application of BOLT-seq

**Fig. 4:**
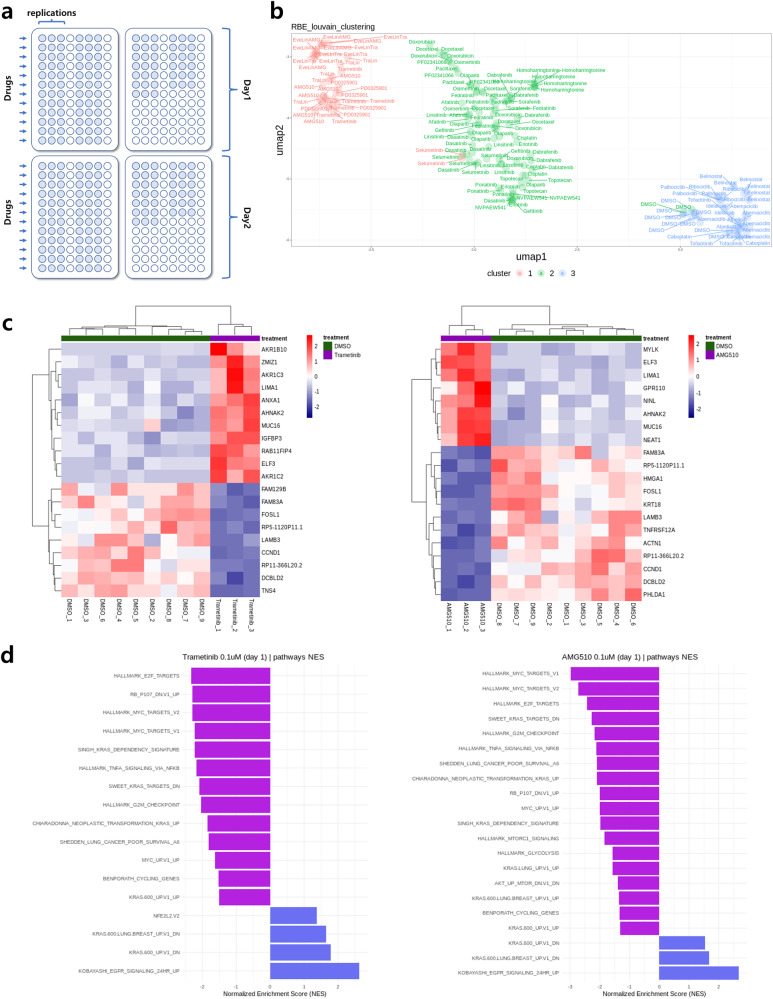
Application of BOLT-seq. **a** Schematic diagram of drug screening with BOLT-seq. **b** Uniform manifold approximation and projection (UMAP) clustering of data for 35 drugs × 2 days × three replicates (DMSO *n* = 9). **c** Top 20 significant DE genes detected in cells exposed to AMG510 or Trametinib. **d** Gene Set Enrichment Analysis for AMG510 and Trametinib.

By continuously adding reagents into a single tube without purifying the reaction products between sequential reaction steps, the cost and time needed for large-scale preparation of NGS libraries can be reduced. Here, a proof-of-concept experiment was performed to show that BOLT-seq can be used in a large-scale screen for drug-induced perturbation of gene expression in NIC-H358 cells, which are KRAS G12C mutant non-small cell lung carcinoma cells. The cells were exposed to 35 drugs [Supplementary Table 3] in triplicate on two experimental days with 9 replicates of the DMSO control, generating 213 data points per sample (Fig. 4a). The NGS library was prepared through the BOLT-seq method, and then gene expression profiling was performed according to the bioinformatic pipelines from the DRUG-seq method^13,31,32^, followed by unsupervised clustering to identify genes with similar drug-induced perturbations^33,34^ (Fig. 4b). Clusters appeared to reflect the type of drug (Supplementary Fig. 5a) but were not influenced by the date of drug treatment (Supplementary Fig. 5b). For example, Cluster 1 included single drugs or drug combinations that target the mitogen-activated protein kinase (MAPK) pathway, which includes the KRAS and MEK proteins. Drugs that inhibit KRAS or MEK inhibit growth and induce apoptosis in NIC-H358 cells^33–35^. AMG510 is a drug that targets KRAS^36^, while trametinib targets MEK^34^, both of which are genes in the MAPK pathway. As AMG510 and trametinib are involved in the same pathway, they are expected to cluster together. To confirm this, we used the DEseq normalization method^21^ and analyzed the normalized and p-adjusted values of gene expression in AMG510- and Trametinib-perturbed samples. Then, the top 20 significant genes for these drugs were identified based on the lowest p-adjusted values (Fig. 4c). BOLT-seq data were also used to identify genes in the ERK pathway^37,38^, such as FOSL1 and CCND1, in cells treated with AMG510 and trametinib. Gene set enrichment analysis (GSEA)^27^ was performed to analyze the perturbation of gene expression in NIC-H358 cells treated with AMG510 or trametinib. The gene set (see Methods) of the results analyzed by GSEA, which is the result of gene expression of NIC-H358 cells treated with ARS1620 and trametinib, was used. GSEA confirmed that AMB510 and trametinib downregulate the expression of KRAS-related genes, including E2F transcription factors, the MYC regulatory network, ERK activation, and KRAS dependency signatures^22–26^ (Fig. 4d).

In summary, BOLT-seq is a novel RNA-seq method that facilitates large-scale transcriptome profiling and drug screening experiments by dramatically reducing the labor, time, and cost of library construction relative to canonical methods. The present study also demonstrates that BOLT-seq performs as well as canonical RNA-seq methods.

## Discussion

Preparing NGS libraries using commercially available kits is labor intensive and very expensive; with the NEBNext Ultra II RNA kit, for example, each sample costs up to US$45–$47. In comparison, one person can generate libraries for up to 96 samples at a time with BOLT-seq, which is complete in 4 h with 2 h of hands-on time and costs US $1.40 per sample. Because BOLT-seq libraries are prepared from unpurified cell lysates using Tn5 transposase-mediated tagmentation of RNA/DNA hybrid duplexes while excluding intermediate purification steps, the time needed for library preparation is dramatically reduced. Furthermore, as M-MuLV RT and Tn5 transposase and their respective buffers are purified in-house, the cost of library preparation is dramatically reduced. In-house-produced M-MuLV RT and Tn5 transposases can be replaced with commercial brand enzymes. However, the final cost of BOLT-seq using commercial brand enzymes is higher than the original BOLT-seq cost, as shown in Supplementary Table 4. As a result, BOLT-seq facilitates large-scale preparation of NGS libraries from unpurified 3′-end mRNA at minimal cost within 4 h; thus, BOLT-seq is a cost- and time-efficient method for large-scale 3′-end mRNA-seq studies.

Comparing the performance of BOLT-seq, TRACE-seq, and NEBNext is challenging. NEBNext is widely used and is the gold standard full-transcriptome mRNA-seq method. Because BOLT-seq is a 3′-end mRNA-seq method, the correlation coefficient for comparing DE genes with BOLT-seq and NEBNext methods (*r* = 0.94) is slightly lower than when TRACE-seq and NEBNext are compared (*r* = 0.96) because TRACE-seq and NEBNext are full-transcriptome mRNA-seq methods. Nevertheless, a correlation coefficient of 0.94 between BOLT-seq and NEBNext is high, given the difference in the methodology.

The performance of BOLT-seq in large-scale drug screening (Fig. 4) is promising. Figure 4 shows successful clustering of drugs by mechanism of action and intended target based on BOLT-seq data. For example, drugs that inhibit MEK and KRAS and downregulate the MAPK pathway inhibit the growth of KRAS-mutant NCI-H358 cells. When a similar screen was performed with NEBNext, more potential drug targets were detected than with BOLT-seq. However, BOLT-seq identified small molecule drug-induced changes in gene expression as well as NEBNext. Due to the relatively lower cost and reduced time needed to prepare and screen libraries with BOLT-seq, 35 different small molecule drugs were screened in triplicate on 2 experimental days. However, the cost of performing the same experiment with NEBNext would likely be prohibitive.

We note that the BOLT-seq method has several limitations and could be improved considerably. First, since BOLT-seq does not cover the whole transcriptome, it is not suitable for studies that focus on alternative splicing events, long noncoding RNAs, or single nucleotide variants in the 5′ end. Nevertheless, reading only the 3′ end plays greatly helps save time and money and provides sufficient information for accurate gene expression measurements. Second, although BOLT-seq costs less than traditional methods, the cost should likely decrease further as the cost of NGS sequencing continues to decrease. Additional cost reduction could be achieved by replacing commercially available DNA polymerase with in-house purified DNA polymerase. Finally, the methodological aspects of BOLT-seq can be improved. Currently, a high percentage of data are discarded when the raw NGS data are preprocessed. Optimizing the BOLT-seq reagents would increase the quality of the cDNA library. Therefore, we hope that the BOLT-seq method will be further improved through future investigation and development.

Much evidence suggests that NGS is an important and effective tool for biomedical research. Because BOLT-seq is a highly scalable, time- and cost-effective, plate-based 3′-end mRNA-seq method, it is expected to support many new and interesting multiscale transcriptomic experiments going forward.

### Supplementary information

Supplementary information
