# Supplementary material for: Cost and time-efficient construction of a 3′-end mRNA library from unpurified bulk RNA in a single tube
Source: Exp Mol Med. 2024 Feb 27;56(2):453–60. doi: 10.1038/s12276-024-01164-8 (PMC10907608; doi:10.1038/s12276-024-01164-8)
Supplement: Supplementary file 1 — Supplementary information [file 12276_2024_1164_MOESM1_ESM.pdf]

Supplementary Figures

a

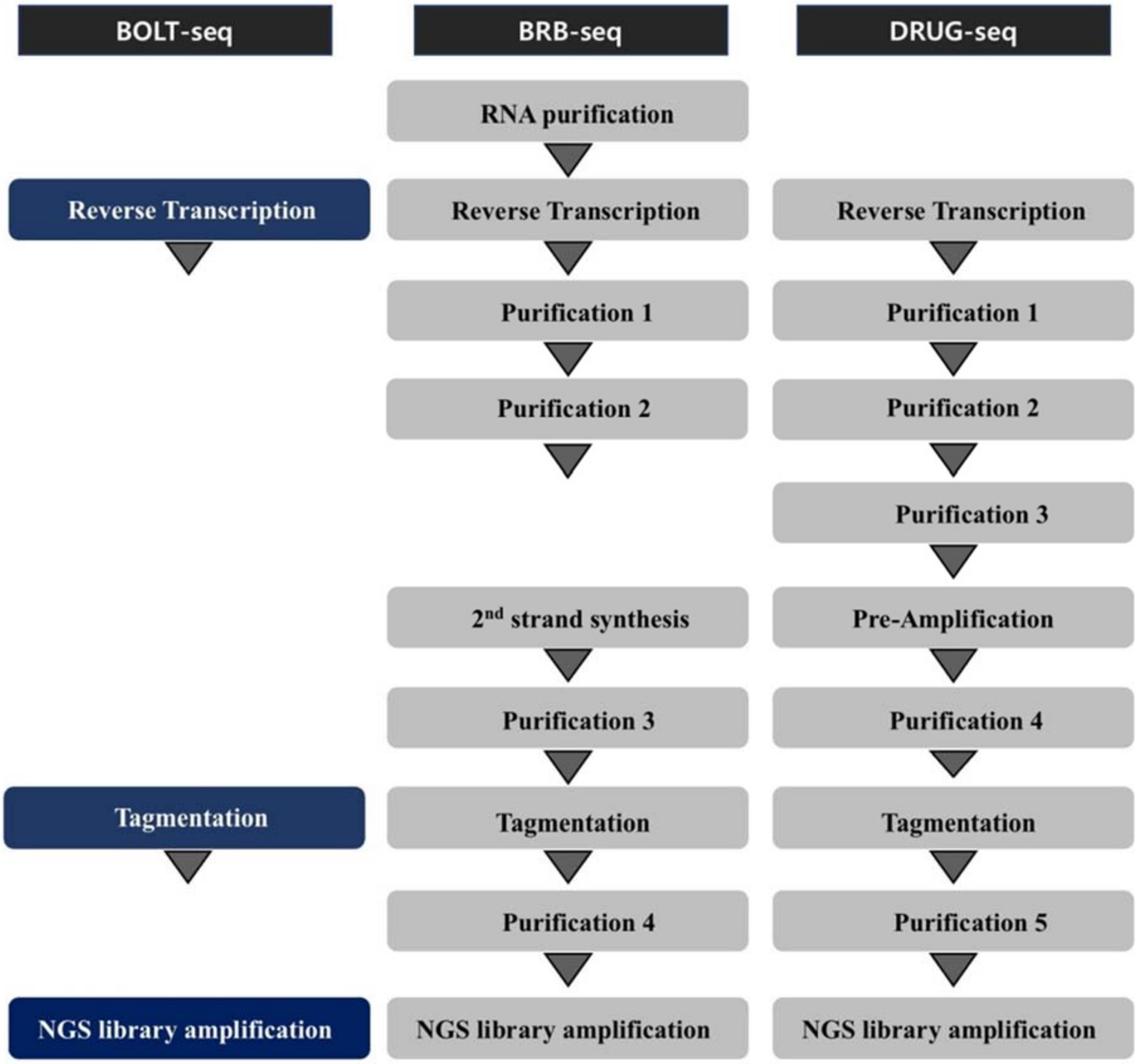

b

	# of cells per well	RNA purification needed	pre-amplification	# of intermediate purification steps	in-house produced products
BOLT-seq	10000	no	no	0	reaction buffers, RT, Tn5
BRB-seq	10000	yes	yes	4	Tn5
DRUG-seq	2500	no	yes	5	none

Supplementary Fig 1. Comparison of different other sequencing methods

(a) Experiment procedure comparison of BOLT-seq with BRB-seq and DRUG-seq. **(b)** Comparison chart of different other sequencing methods.

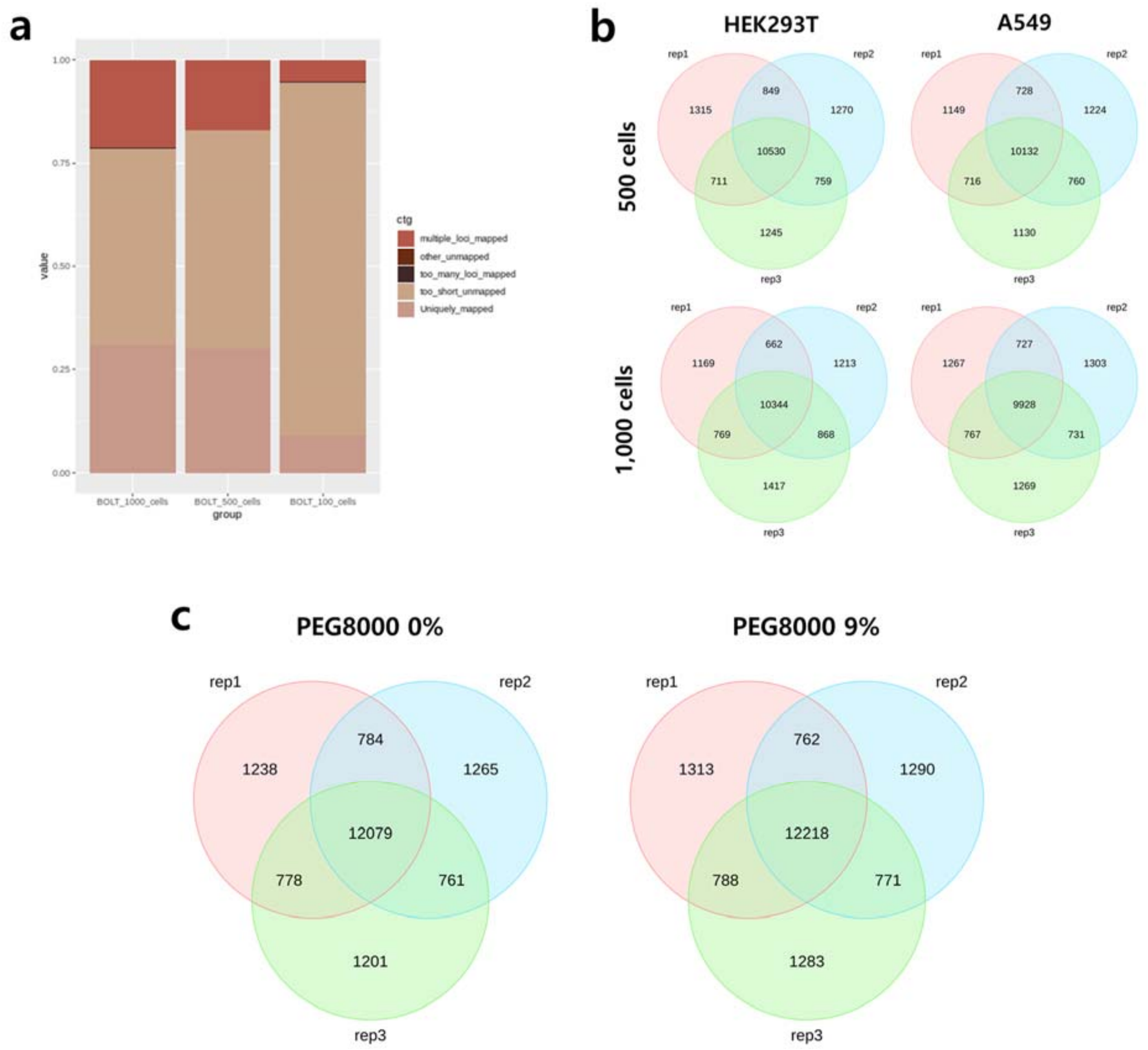

Supplementary Fig 2. Verify experimental conditions of BOLT-seq

(a) Stacked bar graphs represent the number of unique mapped reads using BOLT-seq with 100, 500, or 1,000 cells per well. **(b)** Venn diagrams represent overlapping subsets of genes detected by BOLT-seq using 500 (upper) or 1,000 (lower) cells per well. HEK293T cells, left panels; A549 cells, right panel. **(c)** As in (b) except comparing BOLT-seq in the presence of 0% (left) or 9% (right) PEG8000.

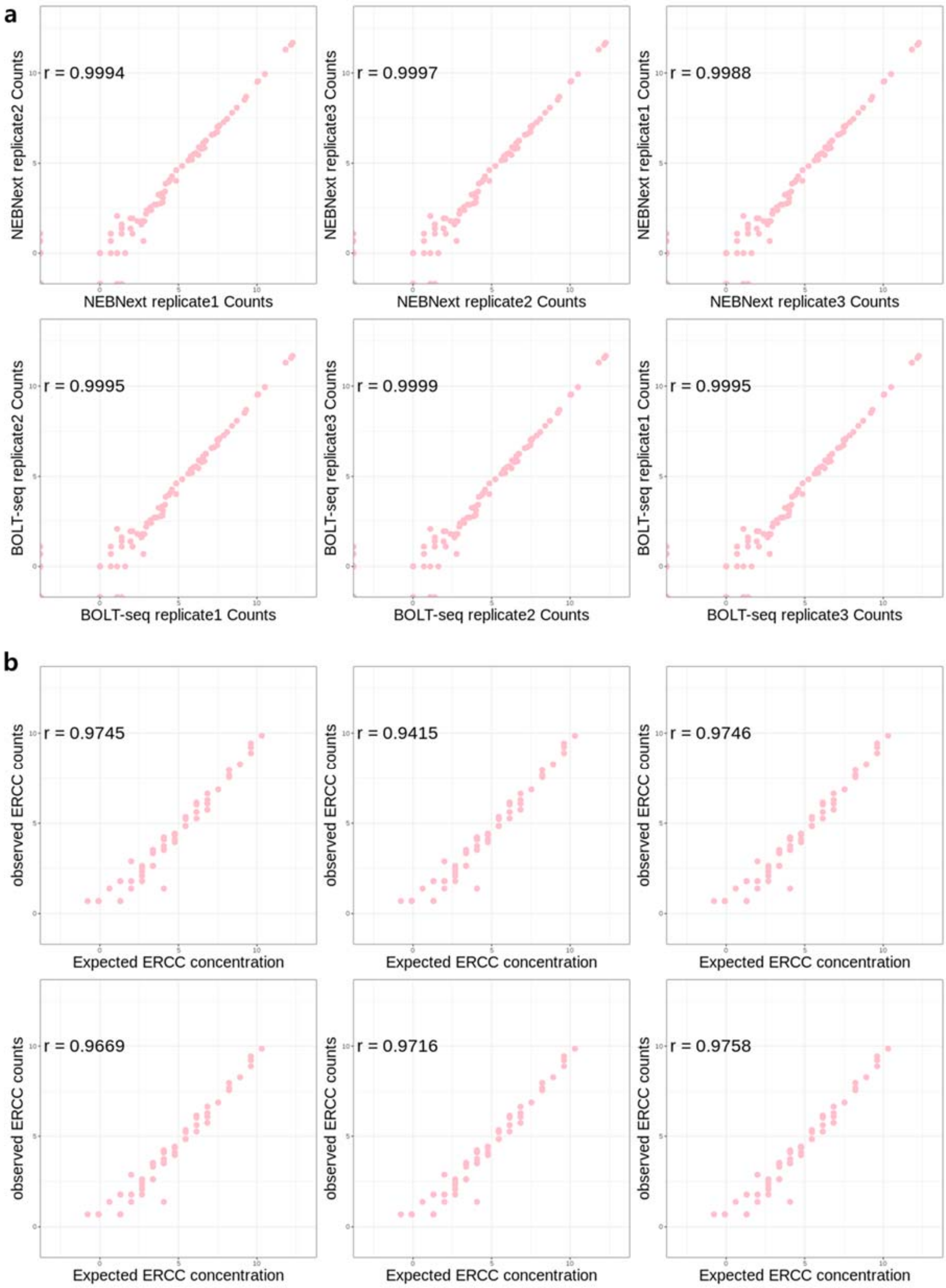

Supplementary Fig 3. ERCC correlations

(a) Correlation between mapped ERCC read counts in three experimental replicates using NEBNext or BOLT-seq. **(b)** Correlation between observed and expected ERCC counts using NEBNext or BOLT-seq.

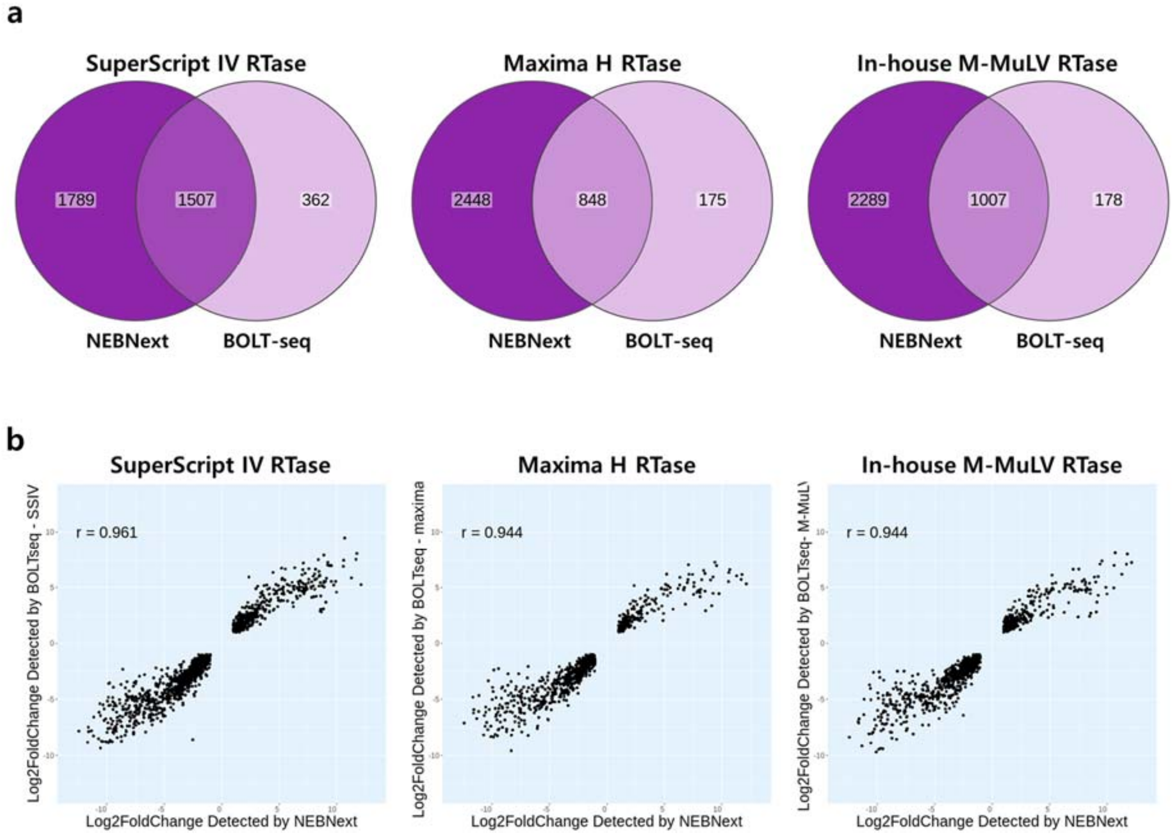

Supplementary Fig 4. Comparison of commercial and in-house reverse transcriptases

(a) Venn diagrams show overlapping subsets of DE genes detected with the NEBNext or BOLT-seq with SuperScriptIV, Maxima-H, or in-house M-MuLV reverse transcriptases (replicates : NEBNext n=3, BOLT-seq n=3). **(b)** Correlation between log2 fold change (lfc) of DE genes with NEBNext or BOLT-seq with different reverse transcriptases as in (a). Threshold: |lfc|>1; p-adj value <0.05.

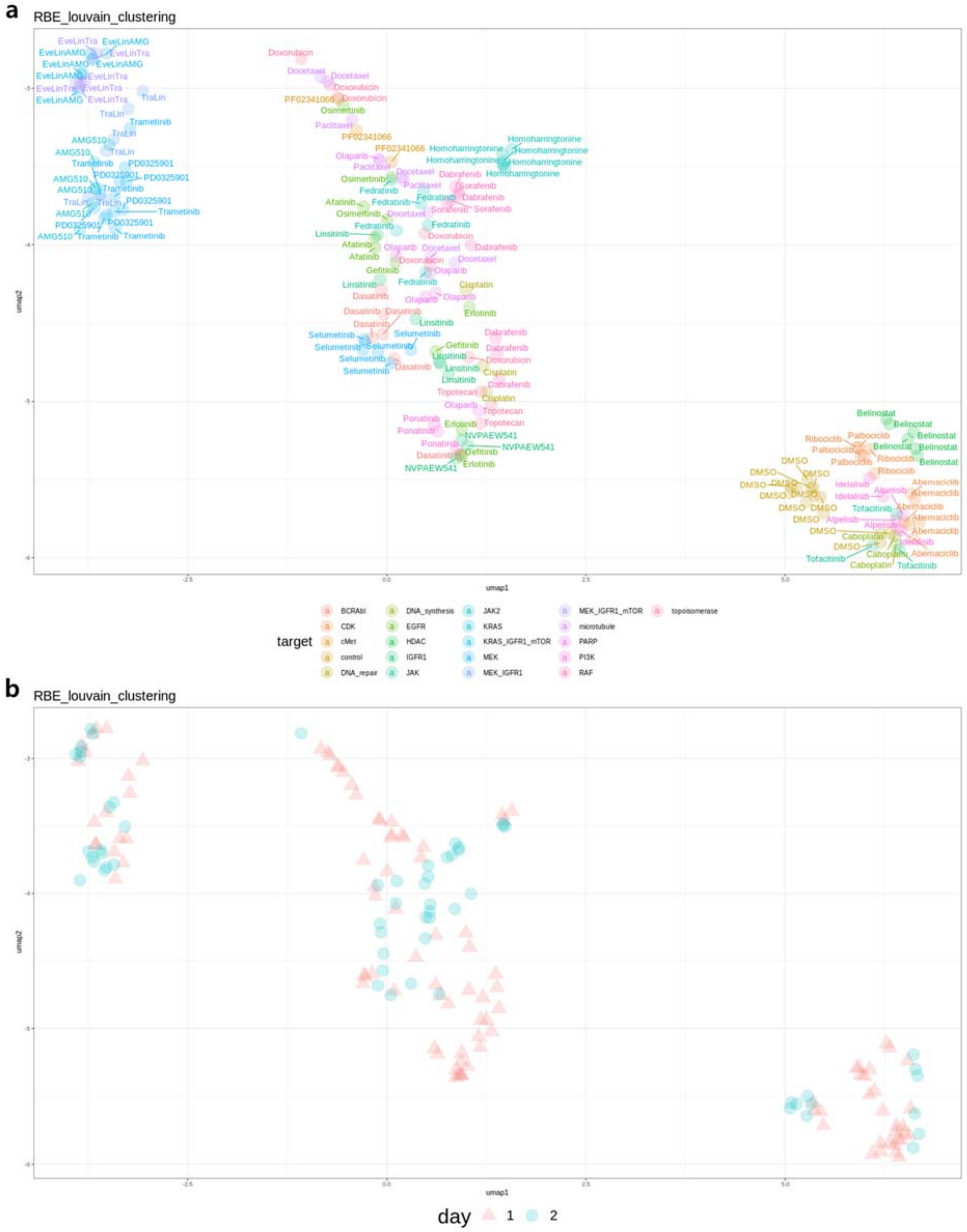

Supplementary Fig 5. UMAP clustering of drug experiment

(a) UMAP clustering by drug target. **(b)** UMAP clustering by experiment day.

Supplementary Tables

Supplementary Table 1. BOLT-seq Price

	BOLT-seq	DRUG-seq	BRB-seq	TM3-seq	TRACE-seq	NEBNext Ultra II RNA kit
Library prep cost per sample	$1.40	$0.2~1.0	$2.4~2.7	$1.50	$12.70	$45~47
Starting material	cell lysate (10,000 cells)	cell lysate (2,500 cells)	purified RNA (50pg-2μg)	purified RNA (200ng)	purified RNA (2ng-200ng)	purified RNA (200ng)
Overall time for library prep	4H	N/A	1 day	6H	6H	1 day
Hands-on time	1~2H	N/A	2H	3H	N/A	N/A
Samples per Run	96	384~1536	96	96	N/A	

Reagent	Manufacturer	Ref	Total units	Units/reaction	Total react	$ Total	$ / reaction	$ per 96 samples
Anchored oligo-dT reverse transcript primers	IDT	X	250 nmol	5 pmol	50,000	54.11	0.0011	0.10
Tn5 oligo	IDT	X	250 nmol	5 pmol	50,000	54.11	0.0011	0.10
dNTP Mix, 10mM each (100ul)	Thermo Scientific	18427-013	10mM/100ul	1mM/ul	1000	98.31	0.098	9.44
Rnase OUT ribonuclease inhibitor	Invitrogen	10777019	5000 units	5 Units	1000	174.53	0.17	16.75
IGEPAL CA-630	Sigma Aldrich	I8896-100ML	100ml	0.18ul	555,555	141.05	0.00025	0.024
PEG8000 (50%)	bioPLUS	40120938-1	500ml	4.3ul	116,279	143.9	0.0012	0.12
Tetraethylene glycol	Thermo Scientific	AC149590010	1000ml	1.0ul	1,000,000	66.2	0.000066	0.0064
KAPA HiFi hotstart	KAPA Biosystems	KK2502	250 units	1 Units	250	156.72	0.63	60.18
SpeedBead Magnetic Carboxylate Modified Particles	GE Healthcare	65152105050350	5000 ml	30ul	166,666	3107.84	0.019	1.79
in-house Reverse Transcriptase	X	X	X	X	X	X	0.30	28.80
in-house Tn5 Transposase	X	X	X	X	X	X	0.15	14.40
Total							1.37	131.72

Supplementary Table 2. Primer Information

Name	Sequence
Anchored oligo(dT)30-P7 RT primer	CGTGTGCTCTTCCGATCTTTTTTTTTTTTTTTTTTTTTTTTTTTTTTTVN
Tn5 P5 oligo	ACACGACGCTCTTCCGATCTNNNNNNNNAGATGTGTATAAGAGACAG

Supplementary Table 3. Drug list and targets

	compound	target
1	Trametinib	MEK
2	Selumetinib	MEK
3	PD0325901	MEK
4	Olaparib	PARP
5	Gefitinib	EGFR
6	AMG510	KRAS
7	NVPAEW541	IGFR1
8	Linsitinib	IGFR1
9	Ponatinib	PI3K
10	TraLin	MEK / IGFR1
11	EveLinAMG	KRAS / IGFR1 / mTOR
12	EveLinTra	MEK / IGFR1 - mTOR
13	Fedratinib	JAK2
14	Homoharringtonine	JAK2
15	Cisplatin	DNA repair
16	Paclitaxel	microtubule
17	Afatinib	EGFR
18	Erlotinib	EGFR
19	Dabrafenib	RAF
20	Osimertinib	EGFR
21	PF02341066	cMet
22	Doxorubicin	topoisomerase
23	Docetaxel	microtubule
24	Dasatinib	BCRAbl
25	Topotecan	topoisomerase
26	Abemaciclib	CDK
27	Palbociclib	CDK
28	Ribociclib	CDK
29	Belinostat	HDAC
30	Alpelisib	PI3K
31	Idelalisib	PI3K
32	Caboplatin	DNA synthesis
33	Tofacitinib	JAK
34	Sorafenib	RAF

Supplementary Table 4. Price Comparison

Reagent	Manufacturer	Ref	Total units	Units/reaction	Total react	$ Total	$ / reaction
Anchored oligo-dT reverse transcript primers	IDT	X	250 nmol	5 pmol	50,000	54.11	0.0011
Tn5 oligo	IDT	X	250 nmol	5 pmol	50,000	54.11	0.0011
dNTP Mix, 10mM each (100ul)	Thermo Scientific	18427-013	10mM/100ul	1mM/ul	1000	98.31	0.098
Rnase OUT ribonuclease inhibitor	Invitrogen	10777019	5000 units	5 Units	1000	174.53	0.17
IGEPAL CA-630	Sigma Aldrich	I8896-100ML	100ml	0.18ul	555,555	141.05	0.00025
PEG8000 (50%)	bioPLUS	40120938-1	500ml	4.3ul	116,279	143.9	0.0012
Tetraethylene glycol	Thermo Scientific	AC149590010	1000ml	1.0ul	1,000,000	66.2	0.000066
KAPA HiFi hotstart	KAPA Biosystems	KK2502	250 units	1 Units	250	156.72	0.63
SpeedBead Magnetic Carboxylate Modified Particles	GE Healthcare	65152105050350	5000 ml	30ul	166,666	3107.84	0.019
BOLT-seq price except inhouse produced enzymes							0.9221

Using in-house produced enzymes	Manufacturer	Ref	Total units	Units/reaction	Total react	$ Total	$ / reaction
in-house Reverse Transcriptase	X	X	X	X	X	X	0.30
in-house Tn5 Transposase	X	X	X	X	X	X	0.15
Total							1.37

Using commercial brand enzymes - 1	Manufacturer	Ref	Total units	Units/reaction	Total react	$ Total	$ / reaction
SuperScript IV Reverse Transcriptase	Invitrogen	18090010	2000 unit	100 unit	20	118.94	5.95
Tn5 Transposase	enzynomics	M065S	20 unit	0.5 unit	40	454.55	11.36
Total							18.23

Using commercial brand enzymes - 2	Manufacturer	Ref	Total units	Units/reaction	Total react	$ Total	$ / reaction
Maxima H Minus Reverse Transcriptase	Thermo Scientific	EP0751	2000 unit	100 unit	20	67.42	3.37
Tn5 Transposase	enzynomics	M065S	20 unit	0.5 unit	40	454.55	11.36
Total							15.66

Using commercial brand enzymesl - 3	Manufacturer	Ref	Total units	Units/reaction	Total react	$ Total	$ / reaction

SuperScript IV Reverse Transcriptase	Invitrogen	18090010	2000 unit	100 unit	20	118.94	5.95
Tn5 Transposase	ABP Biosciences	TN501-1	20ul	0.5ul	40	480.00	12.00
Total							18.87

Using commercial brand enzymes - 4	Manufacturer	Ref	Total units	Units/reaction	Total react	$ Total	$ / reaction
Maxima H Minus Reverse Transcriptase	Thermo Scientific	EP0751	2000 unit	100 unit	20	67.42	3.37
Tn5 Transposase	ABP Biosciences	TN501-1	20ul	0.5ul	40	480.00	12.00
Total							16.29
